# What drug characteristics explain the wide range of manufacturer rebates?

**DOI:** 10.1093/haschl/qxaf132

**Published:** 2025-07-15

**Authors:** Molly T Beinfeld, Fariel LaMountain, Glenn Phillips, Tom Hughes, Peter J Neumann, James D Chambers

**Affiliations:** Center for the Evaluation of Value and Risk in Health, Tufts Medical Center, Boston, MA 02111, USA; Center for the Evaluation of Value and Risk in Health, Tufts Medical Center, Boston, MA 02111, USA; HEOR, Argenx, Inc, Gent 9052, Belgium; HEOR, Argenx, Inc, Gent 9052, Belgium; Center for the Evaluation of Value and Risk in Health, Tufts Medical Center, Boston, MA 02111, USA; Center for the Evaluation of Value and Risk in Health, Tufts Medical Center, Boston, MA 02111, USA

**Keywords:** prescription drug prices, rebates, specialty drugs, health economics

## Abstract

**Introduction:**

Prescription drug list prices, often cited in policy discussions, do not account for rebates negotiated between manufacturers and payers.

**Methods:**

This study examines factors influencing rebates in the United States, focusing on specialty drugs—ie, high priced therapies (often biologics) for which payers issue specific coverage policies. Rebate data came from SSR Health and drug attributes from the Tufts Medical Center Specialty Drug Evidence and Coverage Database. We stratified rebate data by drug type and characteristics.

**Results:**

We identified 161 drugs found in SSR Health and SPEC as of December 2023. We found that rebates vary substantially across drugs (median of 27%, IQR 16%-53%). Biosimilar and originator drugs have the highest, most variable rebates (median of 71%, IQR 53%-79%), while rebates for cancer treatments and orphan drugs are lower (medians of 19% and 23%, respectively) and vary less (IQR of 12%-28% and 14%-29%, respectively). Drugs that face more competition from alternative options within the same therapeutic class, are self-administered, or received Food and Drug Administration approval further in the past have higher rebates.

**Conclusion:**

Our findings indicate that rebates are sizeable and vary along several dimensions, many relating to market competition, complicating policy discussions around drug pricing.

## Introduction

Prescription drug prices in the United States, particularly specialty drugs—high-cost therapies for which payers issue specific coverage policies—have come under increasing scrutiny.^1,2^ Rising list prices are frequently cited as evidence of the economic burden for payers and patients imposed by specialty drugs. However, it is well known that list prices do not reflect the actual prices paid by US payers, who negotiate discounts and rebates with manufacturers in exchange for preferred formulary placement.^3^ The complexity and lack of transparency surrounding rebates create challenges for policymakers trying to understand and address prescription drug affordability for patients.^4^ Additionally, payers and pharmacy benefit managers often fail to pass these negotiated discounts on to patients.^5,6^

Research has shown that rebates vary by therapeutic class—such as insulin products, antidiabetic medications, and tumor necrosis factor inhibitors—but has not examined the potential influence of other factors, including orphan drug status, oncology use, route of administration, recency of approval, and degree of competition.^3^

**Figure 1.**
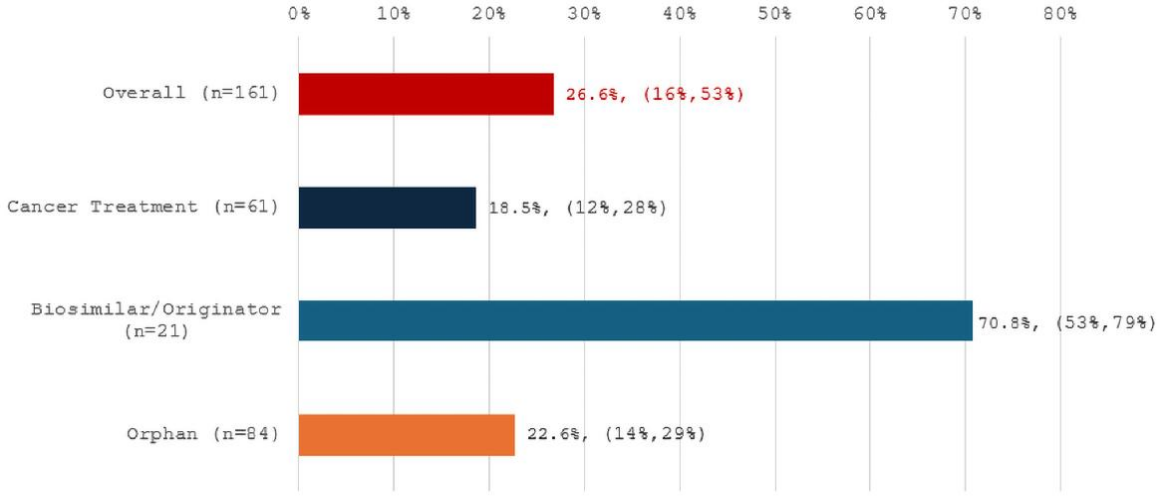
2023 median rebate (%) by drug type. Source: Authors’ analysis of SSR Health, LLC rebate data for 161 specialty therapies, stratified by drug type. Notes: Rebate percentages represent 4-quarter moving averages for calendar year 2023. IQRs are presented in parentheses.

In this study, we examined rebates for 161 specialty drugs in 2023. As shown in Figure 1, the median rebate was 27% with an IQR of 16%-53%. Rebates varied by drug type, being smallest and least varied for drugs treating cancer and orphan diseases, and largest and most varied for biosimilars and originators. Additionally, rebates were larger and more variable for drugs with multiple competitors in the same therapeutic class, those self-administered, or those approved by the Food and Drug Administration (FDA) at least 10 years ago.

## Data and methods

We performed stratified and regression analyses to examine the association between rebates from list price (percent) and 2 key factors: (1) drug type, our primary explanatory variable, and (2) drug characteristics, our secondary exploratory variable.

### Drugs included

To identify “specialty drugs” with specific coverage policies issued by major payers, we focused on pharmaceuticals included in the Tufts Medicine Specialty Drug Evidence and Coverage (SPEC) Database. SPEC includes commercial payer coverage information for 432 specialty drugs as of December 2023 and detailed information on drug attributes such as indications, route of administration, FDA approval dates, and orphan drug status. The SPEC database has been described elsewhere.^7,8^ We further restricted attention to drugs with data available, as of December 2023, from SSR Health, LLC, which tracks rebate information for approximately 1000 brand drugs marketed by 100 currently or formerly publicly traded drug manufacturers. We identified 161 drugs documented by both SPEC and SSR (Appendix 1).

### Rebate data (outcome)

The SSR Health US Brand Rx Net Price Tool contains quarterly list (wholesale acquisition cost [WAC]) and net pricing.^9^ SSR Health compiles data from manufacturer investor reports to determine total revenue after all price reductions (including discounts and rebates) and calculates net prices by dividing the total revenue by the number of prescriptions, estimated by Symphony Health.

This analysis restricted attention to the non-Medicaid pricing data, which excludes statutory Medicaid discounts, from SSR Health. To determine the mean rebate for each drug, we calculated the percentage difference between the reported quarterly WAC and net pricing data and then used these values to compute a 2023 4-quarter moving average percentage rebate for each drug.

### Explanatory variables

Our primary explanatory variable was drug type, categorized as oncology treatment, orphan drug, or biosimilar/originator.

Secondary variables included drug characteristics, including recency of FDA approval (recent: 0-3 years, intermediate: 4-9 years, and older: 10+ years), competition level (defined as the number of drugs within the same therapeutic class approved for the same indication, no competition intermediate: 1-3 alternatives; high: 4+ alternatives), and route of administration (self-administered vs provider-administered). Route of administration was defined by whether the drug is covered under medical or pharmacy coverage. The subset of drugs covered under both plan types was categorized as provider-administered.

### Statistical analysis

For the stratified analysis, we calculated median rebates and IQRs based on drug type (primary factor) and drug characteristics (secondary factor). For the regression analysis, we used ordinary least squares analysis to predict rebate as a function of both drug type and drug characteristics. We conducted analyses using STATA SE17.

#### Limitations

Our sample included drugs produced by publicly traded companies that achieve a minimum sales volume and are featured in a minimum number of coverage policies issued by 18 large commercial payers in the United States. Consequently, our findings may not generalize beyond the specialty drug market (eg, to small molecules, generics, and insulins) or to specialty drugs not included in our sample. Furthermore, the difference in list and net prices includes not only rebates but also other factors such as co-payment cards and Medicare coverage gap mandatory and 340B discounts, which our methodology is not able to distinguish. 340B discounts for specialty drugs, particularly provider-administered specialty drugs, can be substantial. Additionally, because we assigned each drug the same statistical weight in our analysis, rather than weighting them by sales volume, our rebate estimates may not fully represent the overall specialty drug market. Moreover, while we focused our attention on pricing data that excludes Medicaid statutory discounts, estimates from SSR Health still contain supplemental Medicaid rebates. Finally, the included net price data are reported in aggregate and hence may not represent that paid by any individual payer.

## Results

Table 1 presents the distribution of drug types and drug characteristics within our sample of 161 specialty drugs. Percentage rebates for the drugs in our sample varied from 4% to 97%. Appendix 2 lists the variability in rebate estimates by drug characteristics.

**Table 1.** Sample drug characteristics.

	*N*	Percent
Cancer treatment		
Non-cancer treatment	100	62.1
Cancer treatment	61	37.9
Biosimilar/originator		
Non-biosimilar/originator	140	87.0
Biosimilar/originator	21	13.0
Orphan status		
Non-orphan	77	47.8
Orphan	84	52.2
Mode of administration		
Self-administered	66	41.0
Provider-administered	95	59.0
Number of competitors		
No competition (0 alternatives)	15	9.3
Intermediate competition (1-3 alternatives)	60	37.3
High competition (4+ alternatives)	86	53.4
Recency of first indication approval		
Recent approval (0-3 years)	28	17.4
Intermediate approval (4-9 years)	73	45.3
Older approval (10+ years)	60	37.3
ICD-10 code categories^a^		
A00-B99 (certain infectious and parasitic diseases)	3	0.7
C00-D49 (neoplasms)	178	40.0
D50-D89 (diseases of the blood and blood-forming organs and certain disorders involving the immune mechanism)	34	7.6
E00-E89 (endocrine, nutritional and metabolic diseases)	24	5.4
F01-F99 (mental, behavioral, and neurodevelopmental disorders)	5	1.1
G00-G99 (diseases of the nervous system)	28	6.3
H00-H59 (diseases of the eye and adnexa)	14	3.1
I00-I99 (diseases of the circulatory system)	6	1.3
J00-J99 (diseases of the respiratory system)	10	2.2
K00-K95 (diseases of the digestive system)	28	6.3
L00-L99 (diseases of the skin and subcutaneous tissue)	42	9.4
M00-M99 (diseases of the musculoskeletal system and connective tissue)	62	13.9
Q00-Q99 (congenital malformations, deformations and chromosomal abnormalities)	1	0.2
R00-R99 (symptoms, signs and abnormal clinical and laboratory findings, not elsewhere classified)	2	0.4
S00-T88 (injury, poisoning and certain other consequences of external causes)	5	1.1
Z00-Z99 (factors influencing health status and contact with health services)	2	0.4
NA (N/A)	1	0.2

Source: Authors’ analysis of specialty drugs contained in both the Tufts Medical Center's Specialty Drug Evidence and Coverage (SPEC) Database and SSR Health, LLC as of December 2023. ^a^ICD-10 codes for all indications in SPEC database for sample drugs.

### Stratified analyses

**Figure 2.**
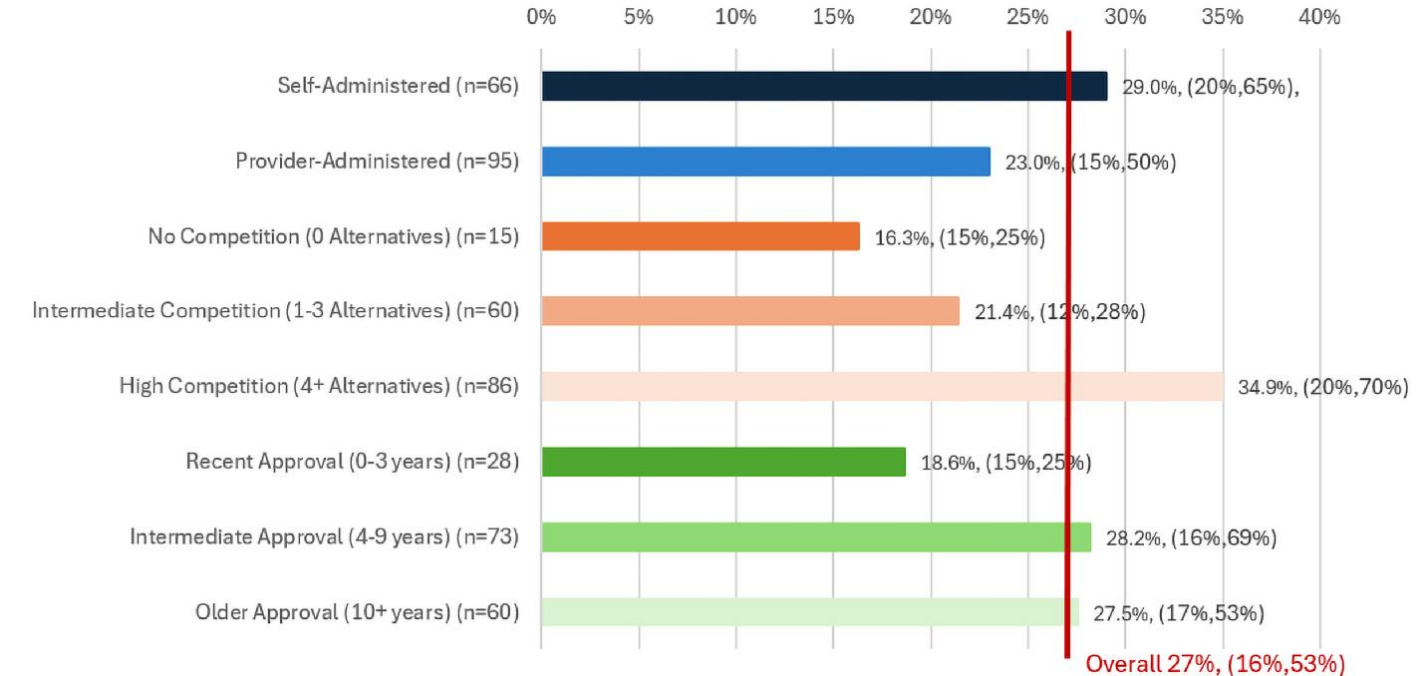
2023 median rebate (%) by drug characteristic. Source: Authors’ analysis of SSR Health, LLC rebate data for 161 specialty therapies, stratified by drug characteristic. Notes: Rebate percentages represent 4-quarter moving averages for calendar year 2023. IQRs are presented in parentheses.

Net prices were lower than list price for all drugs in our sample. For the overall sample, the median drug rebate was 27%, with an IQR of 16%-53%. Figure 1 shows rebate medians and IQRs by drug type. Median rebates for cancer drugs and treatments for orphan indications were generally smaller and less variable compared to the overall sample, whereas rebates for biosimilars and originator products were higher and more variable. Figure 2 illustrates our rebate patterns by drug characteristic, indicating that rebates were smallest and least variable for the most recently approved drugs, those with the least competition, and for provider-administered drugs.

### Multivariate analyses

Table 2 summarizes the results from our regression analysis. We found that larger rebates (*P* < .05) were associated with drugs that were biosimilars or originator products, self-administered, had at least 4 competitors (high competition), or received FDA approval 4-9 years ago. Conversely, factors associated with smaller rebates (*P* < .05) included being a cancer treatment and treating orphan indications. Our multivariate model explained 46% of the variation in rebates.

**Table 2.** Regression results for 2023 4-quarter moving average rebate (%).

Variable	Average rebate (%)
Cancer treatment	−0.117 (3.40)^b^
Biosimilar/originator	0.329 (6.86)^b^
Orphan drug	−0.075 (2.26)^a^
Mode of administration	
Self-administered	Reference
Provider-administered	−0.084 (2.55)^a^
Number of competitors	
No competition (0 alternatives)	Reference
Intermediate competition (1-3 alternatives)	0.071 (1.22)
High competition (4+ alternatives)	0.142 (2.44)^a^
Recency of first indication approval	
Recent approval (0-3 years)	Reference
Intermediate approval (4-9 years)	0.105 (2.47)^a^
Older approval (10+ years)	0.065 (1.45)
_cons	0.268 (3.90)^b^
*R* ^2^	0.453
*N*	161

Source: Authors’ analysis of SSR Health, LLC rebate data for 161 specialty therapies. Rebate percentages represent 4-quarter moving averages for calendar year 2023. ^a^*P* < .05. ^b^*P* < .01.

## Discussion

The findings suggest substantial variation in rebates across specialty drugs, influenced by factors such as therapeutic indication, drug type, mode of administration, competition, and the recency of FDA approval.

As expected, biosimilar and originator drugs had higher and more variable rebates. This aligns with manufacturers’ strategies of offering steeper discounts to capture market share, reflecting patterns seen in the generic drug market.^10^ Similarly, drugs facing higher competition from alternative options approved for the same indication within the same therapeutic class and those with FDA approvals from 4-9 years ago or older had larger rebates, consistent with the need for manufacturers to offer price discounts in competitive markets. Newer drugs in competitive markets may face challenges in gaining market share due to the high rebates offered for existing products. Our finding that difference in net and list prices for provider-administered drugs was lower than that of self-administered drugs is not unsurprising given the limited role of pharmacy benefit managers in contracting for these drugs. Furthermore, these findings might reflect differences in 340B discounts for these drugs rather than rebates. Cancer treatments and drugs for orphan diseases tended to have smaller, less variable rebates, likely due to their narrower market niches and reduced competition. Furthermore, cancer treatments are designated as one of the six protected classes per the Centers for Medicare and Medicaid Services, meaning that part D plans must include the coverage of these drugs and prohibit plans from including prior authorization requirements.^11^ Previous research has demonstrated that this designation limits plans’ ability to negotiate rebates.^12^ These findings, taken together, suggest that various dimensions of competition, independently and collectively, contribute to the variation in rebate size.

Research has shown that higher patient out-of-pocket drug costs are correlated with higher list prices, but not with net prices, suggesting that rebates intended to offset changes in list price are not passed on to patients.^5^ This is problematic because list prices have historically increased faster than inflation and payers have tended to place high cost specialty drugs on specialty formulary tiers with higher coinsurance rates, thereby shifting more costs to patients.^2,13^ Higher list prices may also disproportionately affect uninsured patients or those with high deductibles. Given the magnitude of rebates for specialty drugs and the variation by indication—affecting some patient groups more than others—reforms aimed at equitable access should ensure that patients’ out-of-pocket costs align with actual prices rather than list prices.

Future research should explore the factors driving these relationships and their implications for drug affordability and patient access. The substantial variation observed across drug type and drug characteristic suggests that additional factors may be influencing rebates. The complexity in rebate practices risks creating perverse incentives toward drugs with higher rebates rather than higher value or clinical efficacy. Researchers should also continue to monitor rebate trends, particularly in light of the Inflation Reduction Act (IRA) and its potential impact. For instance, payers and pharmacy benefit managers may have less incentive to prioritize drugs subject to negotiation by the IRA because statutory negotiated Medicare prices will replace rebates for these drugs. Lastly, research should examine the relationship between rebates and specialty drug coverage as it remains unclear whether higher rebates translate into greater patient access to these drugs.

## Conclusion

Our analysis demonstrates that rebates are substantial and vary across drug types and characteristics. Taken together, these drug characteristics point to the crucial role of market competition in determining rebates. However, the unexplained variation in rebates highlights the need for further research in this area. To address prescription drug affordability, reforms should promote greater transparency in rebate practices. Finally, given the magnitude of rebates for specialty drugs and the variation by indication—affecting some conditions more than others—reforms aimed at equitable access should ensure that patients’ out-of-pocket costs align with actual prices rather than list prices.

## Supplementary Material

qxaf132_Supplementary_Data
